# How do leaf anatomies and photosynthesis of three *Rhododendron* species relate to their natural environments?

**DOI:** 10.1186/1999-3110-55-36

**Published:** 2014-03-20

**Authors:** Yan-Fei Cai, Shi-Feng Li, Shu-Fa Li, Wei-Jia Xie, Jie Song

**Affiliations:** 1 Flower Research Institute of Yunnan Academy of Agricultural Sciences, Kunming, 650205 China; 2 Yunnan Flower Breeding Key Lab, Kunming, 650204 China; 3 Yunnan Flower Research and Development Center, Kunming, 650205 China

**Keywords:** Leaf anatomy, Photosynthesis, *Rhododendron*

## Abstract

**Background:**

*Rhododendron* is one of the most well-known alpine flowers. In order to identify performances relating to Rhododendron’s natural habitats we investigated the leaf anatomical structures and photosynthetic characteristics of *R. yunnanense*, *R. irroratum* and *R. delavayi*, which showed different responses after being transplanted into a common environment.

**Results:**

When compared with *R. irroratum* and *R. delavayi*, *R. yunnanense* had lower leaf dry mass per unit area (LMA) and larger stomata, but smaller stomatal density (SD) and total stomata apparatus area percent (At), lower stomatal conductance (Gs), transpiration rate (Tr), light compensation point (LCP), light saturation point (LSP), light-saturated photosynthetic rate (Amax) and leaf nitrogen content per unit area (Na). LMA was positively correlated with Amax and maximum rates of carboxylation (Vcmax). However, leaf N content was not significantly correlated with Amax. Thus, the variation in leaf photosynthesis among species was regulated largely by changes in LMA, rather than the concent of nitrogen in leaf tissue.

**Conclusions:**

*R. yunnanense* plants are vulnerable to moisture and light stress, while *R. irroratum* and *R. delavayi* are better suited to dry and high radiation environments. The present results contribute to our understanding physiological trait divergence in *Rhododendron*, as well benefit introduction and domestication efforts for the three species of *Rhododendron* studied in this work.

**Electronic supplementary material:**

The online version of this article (doi:10.1186/1999-3110-55-36) contains supplementary material, which is available to authorized users.

## Background

*Rhododendron* is one of the most well-known alpine flowers. The genus of *Rhododendron* contains 1000 species distributed in Asia, Europe, and North America. China has the most diverse *Rhododendron* flora with 571 species; 320 species of which occur solely within the Yunnan province of Southwestern China (Fang et al. 2005). However, the distribution of many species is usually narrow in Yunnan province. For example, *R. oxyphyllum* and *R. yunnanense* are mainly distributed in mixed forests on slopes, whereas *R. wardii* and *R. irroratum* are found in evergreen broad-leaved forest or mixed forest, and *R. decorum*, *R. siderophyllum* and *R. delavayi* are found in thickets, hills, rocky slopes or single-species constitute the forest (Fang et al. 2005).

Because of its popularity, the demand of *Rhododendron* genus as an ornamental plant has been increasing in recent years. Large-scale cultivation under controlled conditions is necessary to meet this rising demand. Successful cultivation and continued use of wild species require knowledge of their requirements for optimal growth (Cui et al. 2004). However, few studies have examined the growing conditions of *Rhododendron*, such as water availability, temperature, nutrient availability, and photoprotective cover for overwintering (Cordero and Nilsen 2002; Scagel 2007; Wang et al. 2009).

Photosynthesis is widely used as a tool for indicating environmental stress and selection of growth conditions suitable for different species (Weng and Ueng 1997). The knowledge of leaf structural and physiological characteristics of species and how these triats relate to their physical habitats is essential for introduction and domestication programs (Guan et al. 2011; Zhang and Yin 2012), and subsequent commercial production. Indeed, although several numbers of *Rhododendron* have been cultivated for centuries, it is still not an easy task because the optimum growing condition remains unclear.

Species’ distribution patterns reflect trait-habitat interaction and determined by the plant ecological functions. Plant structure is the basis of function, so the differences and changes of structure will inevitably affect physiological and ecological function, and thus, the adaptability of plants to a changing environment (Kikuzawa 1995; Poorter and Bongers 2006). After transplanting from their natural habitat to the nursery in Kunming without any protection measures, *Rhododendron* species exposed to these different environment (hotter, drier and more radiation) showed divergent responses. For example, *R. yunnanense* grew poorly, with its leaves exhibiting significant light damage, such as leaf chlorosis and sun burn spots, while *R. decorum* and *R. delavayi* maintained normal growth, but with reduced flower size.

The growth and development of plants in such different environments depends on their physiological tolerance, which in turn, relates back to their original habitats. *R. delavayi* and *R. irroratum* are mainly distributed in evergreen broad-leaved forests margins, whereas *R. yunnanense* grow in mixed forests on shaded slopes. Consequently, the most obvious difference of their nature habitat is that *R. delavayi* and *R. irroratum* are subjected to stronger light intensity than is *R. yunnanense* (Fang et al. 2005). We hypothesized that the difference in light intensity influences the distribution of these species of Rhododendron.

Light is one of the most important driving forces of leaf photosynthesis, which in turn determines a plant’s growth, survival and fitness. In response to changes in light environment, plants acclimate to different light availabilities mainly through changes in leaf anatomical features (particularly changes in leaf mass per unit area, LMA), or by changes in biochemical features (particularly leaf nitrogen content and leaf nitrogen partitioning among different photosynthetic machinery), or both (Feng 2008; Evans and Poorter 2001; Lin and Hsu 2004). However, ecological studies have generally focused on anatomical features without distinguishing changes in leaf nitrogen content, and physiological studies have concentrated on the importance of biochemical changes without considering the importance of changes in structure (Le Roux et al. 2001). The relative importance of these anatomical and physiological variations within a given plant material is not well understand (Le Roux et al. 1999).

The leaves of a plant are the main apparatus for photosynthesis and respiration, and closely link with the surrounding environment. In the evolution of plants, leaves are the most sensitive organs and the plasticity is the largest to the environment. Leaf structural features contributing to the maintenance of the high CO_2_ concentration in the chloroplast stroma may have been selected during evolution (Dunbar-Co et al. 2009). Therefore, the research for leaf traits indicative of leaf performance is crucial to understanding of the ecological function and the distribution of plant species (Vendramini et al. 2002; Pandey et al. 2009).

In this study, we investigate leaf anatomical structure and photosynthetic characteristics of three *Rhododendron* species that showed different responses when grown under the same environment. Our aim was to identify their divergent performances relating to natural habitat, and evaluate the relative importance of leaf anatomy and physiology in relation to their natural habitats. The relationships between leaf anatomy and physiological aspects are particularly emphasized.

## Methods

### The study site and plant materials

Three year old plants of *R. yunnanense*, *R. irroratum* and *R. delavayi* were collected from their natural habitats in the east of Yunnan province, China (alt. 1500-2409 m, E 103°42′-104°34′, N 24°20′-25°00′). This site has a subtropical mountain climate, and the main vegetation is conifer and broad-leaved mixed forest. The ecological characteristics and biological traits considered are shown in Table 1 in Fang et al. (2005). Three *Rhododendron* species were cultivated in a nursery in Kunming, China (a1t, 1926 m, E 102°46′, N 25°07′) after collected them from the field. The mean annual temperature and mean annual rainfall were 14.5°C and 1035.3 mm, respectively. The seedlings were grown into 3-L plastic pots (one plant per pot) filled with a laterite-humus (V/V, 1/3), shaded by a nylon net to give 40-50% of full sunlight, and then watered and fertilized as needed to ensure non-limiting water and nutrient supply. After cultivation for 18 months, the plants were used for measurements in the present study.

**Table 1:** **Ecological characteristics and biological traits of three** ***Rhododendron*** **species**

Species	***R. yunnanense***	***R. irroratum***	***R. delavayi***
Life form	Shrubs, rarely small trees	Shrubs or small trees	Shrubs or trees
Distribution	Guizhou, S Shanxi, W Sichuan, Xizang, Yunnan	W Guizhou, SW Sichuan, N and SE Yunnan	NW Guangxi, W Guizhou, SW Sichuan, SE Xizang, Yunnan
Altitude (m)	2200-3600	1700-3500	1200-3200
Habitat	Mixed forests on shade slopes, Abies-Picea or Pinus-Quercus forest margins, thickets	Evergreen broad-leaved forests, mixed forests	Mixed forests, evergreen broad-leaved forests, forest margins, thickets, hills, rocky slopes, open field
Flower period	Apr-Jun	Mar-May	May
Fruit period	Sep-Oct	Sep-Oct	Dec

### Gas exchange measurements

Gas exchange measurements were made on the fully expanded, mature leaves using a portable LI-6400XT photosynthesis system (LI-Cor, Lincoln, NE, USA) equipped with a red/ blue LED light source (6400-02B) in July 2010. The response curve of net photosynthetic rate (A_n_) to irradiance (PAR) was determined at light intensities (0–1600 μmol m^−2^ s^−1^). During measurements, leaf temperature (T_leaf_) was controlled at 20°C, CO_2_ concentration (C_a_) and relative humidity (RH) in the cuvette were held at 370 μmol mol^−1^ and 60-70%, respectively. A_n_-C_i_ curve was determined with a range of CO_2_ concentrations (0–2000 μmol mol^−1^) under a controlled light intensity (800 μmol m^−2^ s^−1^). T_leaf_ and RH were controlled as the same with A_n_-PAR curves. Three mature leaves from three individual plants per species were selected for photosynthetic measurement. A_n_, stomatal conductance (G_s_) and transportation rate (T_r_) were recorded automatically during measurements. Water use efficiency (WUE) was calculated as dividing A_n_ by T_r_. Values of G_s_ and WUE were measured at C_a_ of 370 μmol mol^−1^ and PAR of 800 μmol m^−2^ s^−1^. Light-saturated photosynthetic rate (A_max_), dark respiration rate (R_d_), light saturation point (LSP) and light compensation point (LCP) were calculated from the A_n_-PAR curve with a three-component exponential function (Watling et al. 2000): A_n_ = a(1-e^-bx^) + C, where A_n_ is photosynthetic rate, x is PAR, a, b and C are constants. Maximum rates of carboxylation (V_cmax_) was estimated from the A_n_-C_i_ curve with Photosyn Assistant software (Dundee Scientific, Scotland, UK) that applied the biochemical model described by Von Caemmerer and Farquhar (1981).

### Leaf properties and anatomical measurements

After the gas exchange measurements, 9 mature leaves from 3 individual plants for each species were harvested for subsequent analysis. Chlorophyll content was determined using a spectrophotometer UV-751GD (Shanghai Analytical Instrument Co., China) and was calculated according to the method of Inskeep and Bloom (1985). Leaf area was measured with a leaf area meter LI-3000A (LI-COR, Inc., Nebraska, USA). Leaf dry weight was obtained after oven-drying at 80 for 48 h. Leaf dry mass per unit area (LMA) was calculated using these data. The dried leaf samples used for LMA measurement were collected and use for leaf nitrogen content per area (N_a_) analyses with an automatic elemental analyser (Vario EL, Elementar Analysensysteme GmbH, Hanau, Germany). The partitioning coefficients for leaf nitrogen in RuBPCO (P_R_) and bioenergetics (P_B_) were estimated according to the method of Niinemets and Tenhunen (1997). The values of P_R_ and P_B_ were calculated as follows:

PB=Jmax8.06NmJmcLMAPR=Vcmax6.25NmVcrLMA

The values of V_cr_ and J_mc_ at 20°C were equal to 12.6 μmol (CO2) g^−1^ (RuBPCO) s^−1^ and 131.9 mol (electron) mol^−1^ cyt f s^−1^, respectively. N_m_ was mass-based leaf nitrogen content (%). The PNUE was calculated as A_max_ / N_a_.

Small pieces from the middle region of the leaves were fixed in FAA (formalin/ glacial acetic acid/ 50% ethanol, V/ V/ V, 5/ 5/ 90) for at least 24 h, then dehydrated by gradient ethanol, cleared in xylene, and then embedded in paraffin for sectioning. 8-μm thick sections were cut using a Microm HM 315 rotary microtome (Microm Laborgeraet GmbH, Germany) and then mounted on glass slides. The samples were examined and photographed using a Nikon Eclipse E800 light microscope (Nikon, Melville, NY, USA). Cuticle, epidermis, mesophyll, palisade tissue, spongy tissue and leaf thickness were measured with image ProPlus 6.0 software at 400× magnification (Media Cybernetics, Inc., Sliver Spring, MA, USA).

Small sections cut out from the middle section of leaves (ca. 0.5 × 0.5 cm) were placed in a 50% sodium hypochlorite solution until the leaves turned white, and then peeled adaxial and abaxial epidermis after washing with distilled water and photographed under a light microscope. Digital images were analyzed with ProPlus 6.0 software at 400× magnification. The stomatal density (S_D_), stomatal apparatus area (A_s_) and total stomatal apparatus area percent (A_t_) were calculated as follows: S_D_ = N/ S, A_S_ = 1/4 × π × l × w, A_t_ = A_S_ × S_D_ × 100%. N denotes the number of stomata, S is the area of observation, and l and w are the stomatal apparatus length and width. Three leaves from three individuals were examined for each species, and more than ten images per leaf were analyzed.

### Statistical analysis

Statistical analysis was performed with SPSS 13.0 (SPSS Inc., Chicago, USA). Difference between means were tested by one-way ANOVA and LSD multiple comparisons tests. Difference was considered significant at p < 0.05. All graphs were carried out in the software SigmaPlot for windows version 9.0 (Systat Software, Inc.).

## Results

### Leaf anatomical structure

Leaf thickness (LT) of three *Rhododendron* species varied from 243.98 to 262.42 μm, with *R. yunnanense* having the thickest leaves (262.42 μm). Adaxial epidermis cell thickness (ET_ad_), abaxial epidermis cell thickness (ET_ab_) and mesophyll tissue thickness (palisade and spongy tissue thickness, PT and ST) contributed to higher leaf thickness of *R. yunnanense* (Table 2). However, cuticle thickness (CT) and ratio of palisade and spongy tissue (PT/ST) were lower in *R. yunnanense. R. delavayi* had the lowest LT and highest PT/ST, whereas *R. irroratum* was in the middle (Table 2).

**Table 2:** **Leaf anatomical structure and stomatal characteristics of three** ***Rhododendron*** **species**

Parameters	***R. yunnanense***	***R. irroratum***	***R. delavayi***
LT (μm)	262.42 ± 0.98a	247.47 ± 1.63b	243.98 ± 2.57b
CT (μm)	2.76 ± 0.12c	5.93 ± 0.11a	3.76 ± 0.10b
ET_ad_ (μm)	40.09 ± 0.46a	39.45 ± 0.32a	35.46 ± 0.46b
ET_ab_ (μm)	13.93 ± 0.33a	7.98 ± 0.11b	7.18 ± 0.14c
PT (μm)	91.53 ± 0.80a	84.31 ± 0.59b	91.35 ± 2.03a
ST (μm)	114.94 ± 1.03a	109.86 ± 1.50b	105.30 ± 1.83b
PT/ST	0.81 ± 0.01b	0.78 ± 0.01b	0.89 ± 0.03a
S_D_ (mm^2^)	198.56 ± 4.20c	501.00 ± 11.17b	810.99 ± 15.32a
l (μm)	17.99 ± 0.32a	11.74 ± 0.15b	10.22 ± 0.18c
w (μm)	11.06 ± 0.20a	7.77 ± 0.12b	6.40 ± 0.15c
A_s_ (μm^2^)	620.44 ± 13.98a	345.26 ± 5.02b	243.17 ± 5.66c
A_t_ (%)	11.07 ± 0.28c	18.09 ± 0.33b	21.03 ± 0.50a

Mean ± SE (n = 50) LT, leaf thickness; CT, cuticle thickness; ET_ad_, adaxial epidermis cell thickness; ET_ab_, abaxial epidermis cell thickness; PT, palisade tissue thickness; ST, spongy tissue thickness; PT/ST, ratio of palisade and spongy tissue; S_D_, stomatal density; L, stomatal length; W, stomatal width; A_s_, stomatal apparatus area; A_t_, total stomatal apparatus area percent. Different letters in the same row indicate statistical difference (p < 0.05).

**Figure 1:**
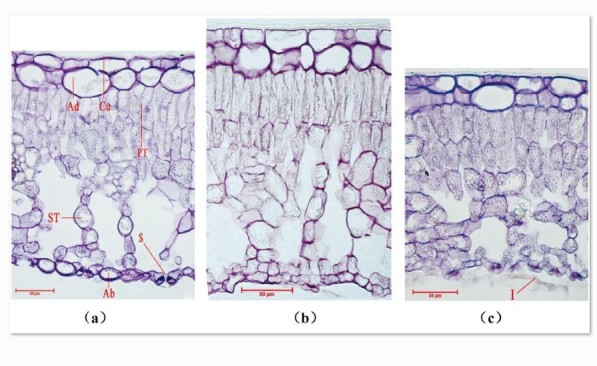
**Leaf cross sections of** ***Rhododendron yunnanense*** **(a),** ***R. irroratum*** **(b), and** ***R. delavayi*** **(c) under light microscope.** Cu, cuticle, A_d_, adaxial epidermis, PT, palisade tissue, ST, spongy tissue, A_b_, abaxial epidermis, S, stomata. I, indumentum. Scale bars 50 μm.

**Figure 2:**
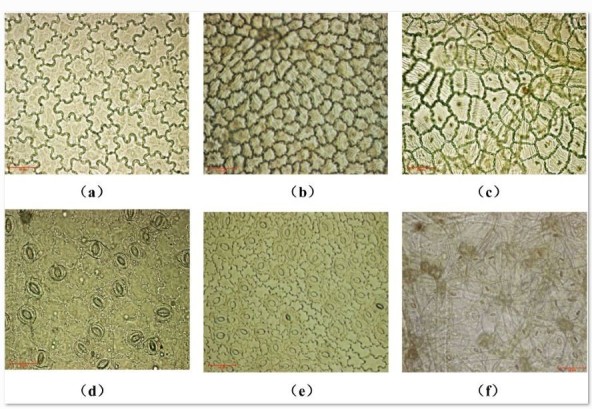
**Adaxial epidermis (a-c) and abaxial epidermis (d-f) of** ***Rhododendron yunnanense*** **(a, d),** ***R. irroratum*** **(b, e), and** ***R. delavayi*** **(c, f) under light microscope.** Scale bars 50 μm.

Adaxial epidermal cells of three *Rhododendron* species are rectangular or ellipse and closely arranged in two layers of cell (Figure 1). The shape of epidermal cells of *R. yunnanense* and *R. irroratum* were wavy (Figure 2a and b), whereas *R. delavayi* had polygon-shaped epidermal cells (Figure 2c). Oval stomata were only found on the leaf abaxial surface among the three *Rhododendron* species (Figure 2). There were significant differences in stomatal characteristics among the considered species. The stomatal density (S_D_) and total stomatal apparatus area percent (A_t_) were lowest in *R. yunnanense*, and highest in *R. delavayi*. Stomatal length (l), stomatal width (w) showed opposite trends with S_D_ and A_t_ (Table 2).

### Leaf photosynthetic capacity

*R. delavayi* and *R. irroratum* had higher A_max_, LCP, LSP, R_d_, and V_cmax_, and were significantly different from *R. yunnanense* (p < 0.05) (Table 3). However, there were no significant differences between *R. delavayi* and *R. irroratum*. Furthermore, *R. yunnanense* had the lowest intercellular CO_2_ concentration (C_i_) at atmospheric CO_2_ concentration of 370 μmol mol^−1^ among the three species (Table 3), possibly a result of lower G_s_ (0.144 mol m^−2^ s^−1^). Lower G_s_ also resulted in lower T_r_ in *R. yunnanense*, and therefore improved water use efficiency (WUE). *R. delavayi* and *R. irroratum* showed opposite trend with *R. yunnanense,* and with higher values of G_s_, C_i_, T_r_ and lower value of WUE.

**Table 3:** **Leaf gas exchange parameters (Mean ± SE) of three** ***Rhododendron*** **species**

Parameters	***R. yunnanense***	***R. irroratum***	***R. delavayi***
A_max_ (μmol m^−2^ s^−1^)	9.00 ± 0.38b	11.19 ± 0.99a	11.90 ± 0.19a
LCP (μmol m^−2^ s^−1^)	5.16 ± 0.63b	8.67 ± 1.10a	10.84 ± 0.69a
LSP (μmol m^−2^ s^−1^)	509.55 ± 60.15b	643.68 ± 70.34a	665.37 ± 8.19a
R_d_ (μmol m^−2^ s^−1^)	0.35 ± 0.04b	0.64 ± 0.11a	0.77 ± 0.03a
V_cmax_ (μmol m^−2^ s^−1^)	29.00 ± 1.00a	32.00 ± 1.00a	32.67 ± 1.45a
C_i_ (μmol mol^−1^)	251.89 ± 9.09b	273.94 ± 2.49a	281.20 ± 5.47a
G_s_ ( mol m^−2^ s^−1^)	0.144 ± 0.015b	0.232 ± 0.024a	0.292 ± 0.028a
T_r_ ( mmol m^−2^ s^−1^)	1.46 ± 0.09c	2.18 ± 0.17b	3.00 ± 0.30a
WUE (μmol CO_2_ mmol H_2_O^−1^)	5.90 ± 0.31a	4.83 ± 0.09b	3.81 ± 0.37c

Amax, light- saturated photosynthetic rate; LCP, light compensate point; LSP, light saturate point; Rd, dark respiration rate; Vcmax, maximum rate of RuBP carboxylation; Gs, stomatal conductance; Ci, intercellur CO2 concentration; Tr, transpiration rate; WUE, photosynthetic water use efficiency. Different letters in the same row indicate statistical difference (p < 0.05).

### Leaf properties

LMA of *R. irroratum* was significantly higher than *R. yunnanense*, but similar to that of *R. delavayi* (Table 4). *R. yunnanense* had the lowest value of N_a_ and highest value of N_m_, while P_R_, P_B_ and PNUE were highest in *R. delavayi*, but there was no significant difference among three *Rhododendron* species except for N_m_. *R. irroratum* had the highest value for chlorophyll content per area (Chl), but the lowest ratio of chlorophyll a to b (*Chl* a*/* b).

**Table 4:** **Leaf traits of three** ***Rhododendron*** **species**

Parameters	***R. yunnanense***	***R. irroratum***	***R. delavayi***
LMA (g m^−2^)	59.38 ± 1.90b	94.32 ± 2.97a	87.08 ± 3.33a
N_a_ (g m^−2^)	0.98 ± 0.05a	1.20 ± 0.12a	1.01 ± 0.09a
N_m_ (mg g^−1^)	16.50 ± 1.18a	12.69 ± 1.21ab	11.67 ± 1.15b
P_R_ (g g^−1^)	0.378 ± 0.008a	0.344 ± 0.023a	0.414 ± 0.029a
P_B_ (g g^−1^)	0.088 ± 0.001a	0.087 ± 0.010a	0.095 ± 0.006a
PNUE (μmol g^−1^ s^−1^)	9.26 ± 0.59a	9.44 ± 0.85a	11.92 ± 0.97a
*Chl* a + b (μg m^−2^)	62.91 ± 2.85a	66.05 ± 4.18a	57.58 ± 4.87a
*Chl* a/ b	2.43 ± 0.06ab	2.25 ± 0.52b	2.53 ± 0.08a

LMA, leaf mass per unit area; Na, leaf nitrogen content per unit area; Nm, leaf nitrogen content per unit mass; PR, the partitioning coefficients of leaf nitrogen in RuBPCO; PB, the partitioning coefficients of leaf nitrogen in bioenergetics; PNUE, photosynthetic nitrogen use efficiency; *Chl* a+b, chlorophyll content of a and b; *Chl* a/ b, the ratio of chlorophyll a and b. Different letters in the same row indicate statistical difference (p < 0.05).

### Relationships between leaf traits

**Figure 3:**
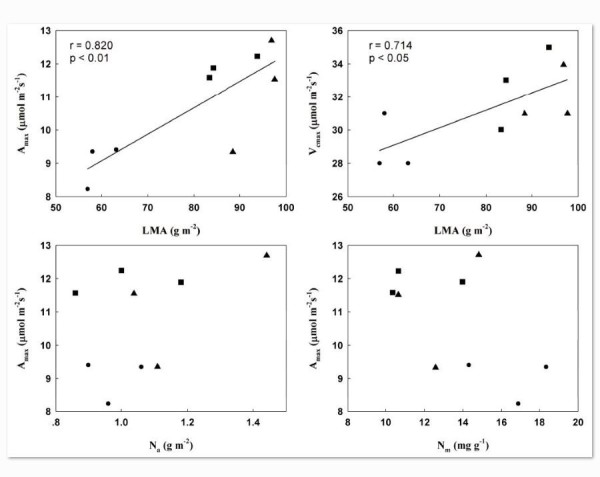
**Correlations between leaf dry mass per unit area (LMA) and light-saturated photosynthetic rate (A**_**max**_**) or maximum rates of carboxylation (V**_**cmax**_**); and between A**_**max**_**and leaf nitrogen content per unit area (N**_**a**_**) or leaf nitrogen content per unit mass (N**_**m**_**) in three** ***Rhododendron*** **species.** “●”, “▲” and “■” stand for *R. yunnanense, R. irroratum* and *R. delavayi*, respectively.

**Figure 4:**
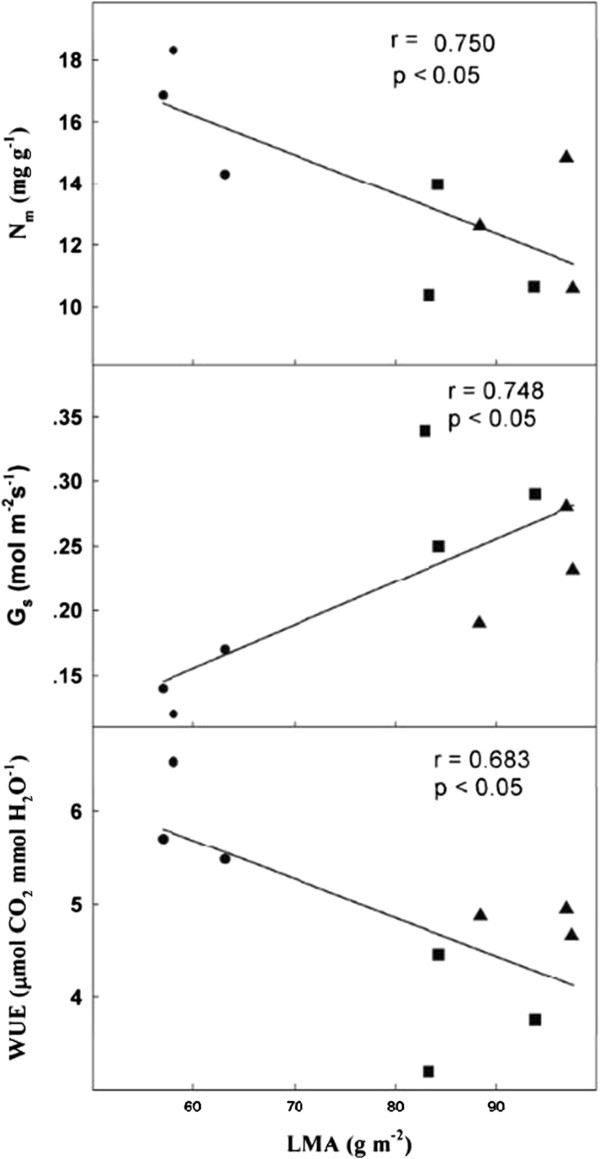
**Correlations between leaf dry mass per unit area (LMA) and leaf nitrogen content per unit mass (N**_**m**_**), stomatal conductance (G**_**s**_**) and water use efficiency (WUE) in three** ***Rhododendron*** **species.** “●”, “▲” and “■” stand for *R. yunnanense, R. irroratum* and *R. delavayi*, respectively.

There was a strong linear correlation between LMA and photosynthetic capacity, as estimated by A_max_ and V_max_, although there was no significant relationship between A_max_ and N content either base on leaf area or leaf mass (Figure 3). In addition to relating with photosynthetic capacity, LMA showed a negatively correlation with N_m_ or WUE, and a positive correlation was found between LMA and G_s_ (Figure 4).

## Discussion

### Correlation between leaf structure and natural habitat

Plants often exhibit considerable variations in their functional traits that affect the capture and utilization of resources and enable them to adapt to changing environments (Guan et al. 2011). Cuticles can reduce water loss from the leaf to the atmosphere, and considered as an ecological characteristic for plant confront to high light and drought. The cuticle of *R. irroratum* was thicker than *R. yunnanense*, whereas *R. delavayi* did not have a thicker cuticle than *R. irroratum*. However, the abaxial surface of *R. delavayi* with 1-layered spongy, or somewhat agglutinated indumentum (Figure 2f), can reduce water loss from the leaf interior and prevent light damage, and thus increase water use efficiency and maintain normal leaf physiological function (Mill and Stark Schilling 2009). The results indicate *R. yunnanense* is vulnerable to moisture and light stress, whereas *R. irroratum* and *R. delavayi* may be better suited to drier and brighter habitats.

Stoma effectively regulates gas exchange where water vapor leaves the plant and CO_2_ enters. The stomatal distribution, density, size, morphology and behavior are closely associated with environmental factors (Schlüter et al. 2003). The significantly negative correlation found between stomatal size and stomatal density in three *Rhododendron* species, i.e., *R. delavayi* with more densely but smaller stomata and *R. yunnanense* showed opposite trends (Table 2). Stomatal density is closely related to water availability and light intensity (Cai et al. 2004). Generally speaking, strong light and water deficit lead to more densely but small stomata (Xu and Zhou 2008). Small stomata enable the leaf to attain high and rapid diffusive conductance under favorable conditions, and they afford greater water use efficiency in dry habitats because they can react more quickly to environmental stimuli (Franks et al. 2009). By contrast, larger stomata are slower to close and have a greater potential for hydraulic dysfunction under conditions of drought, however, this lag in response may be advantageous in cool, moist, or shaded environments (Aasamaa et al. 2001). The results indicated that *R. delavayi* with more densely but smaller stomata may have a strong ability to regulate water/CO_2_ and may be better suited to more arid and high light environments than are *R. yunnanense* or *R. irroratum*. This is consistent with our observation of these species’ natural habitats.

LMA is a key structural trait that measures plant investment and is widely used as an indicator of plant ecological strategies (Westoby et al. 2002). A high LMA was associated with high leaf thickness and more structural tissue, and has been interpreted as a property to drought or high light environment (Salleo and Lo Gullo 1990). However, in present study, LMA didn’t show a positive correlation with leaf thickness. LMA of *R. yunnanense* was significantly lower than *R. delavayi* and *R. irroratum* (Table 4), but its leaf was thicker. Leaf mesophyll (palisade and spongy tissue) mainly contributed to a thicker leaf (Table 2). Witkowski and Lamont (1991) point out that both leaf thickness and density may account for changes in LMA and both traits may vary independently. Moreover, anatomical structure underlying variation in leaf density and thickness may differ depending on the nature of the species and their environment (Garnier and Laurent 1994; Van Arendonk and Poorter 1994; Poorter et al. 2009). Castro-Díez et al. (2000) found LMA in 52 European woody species was correlated with leaf density but not with thickness. Greater LMA across these species set as greater allocation to support and defense functions, as shown predominantly by species from resource-poor environment.

### Correlation between leaf physiological function and natural habitat

Light has been justified as the main factor determining a plant’s survival, growth, and fitness. Upon exposure to a wide range of light regimes, plants show an ability to meet these differing conditions, mainly by alterations in leaf structure and biochemistry (Evans 1989; Niinemets and Tenhunen 1997). LCP and LSP of *R. delavayi* and *R. irroratum* were higher than *R. yunnanense*, indicating their photosynthetic apparatus can operate well in higher light environment and benefiting to survival and occupy higher light habitats. The study of Nilsen et al. (1988) about *R. maximum* also reported that in the open environment, the rate of light saturated photosynthesis was reached earlier in the day than that of low light environments. For shade-tolerant *R. yunnanense*, lower LCP, LSP, and Rd would benefit greater net carbon gain in low-light, and suggest this species may be a strong competitor in low-light environments.

Because more than 50% of total leaf nitrogen is allocated to the photosynthetic apparatus, total leaf nitrogen content affects the biochemical efficiency of assimilation (Evans 1989). Generally, the leaves on plants grown under low light have more nitrogen content per mass than those exposed to high light, this is because shade leaves contain less mechanical tissue per unit area than do sun leaves (Evans and Poorter 2001). Our studies also yielded similar results. *R. delavayi* and *R. irroratum* had higher N_a_ than *R. yunnanense*, whereas N_m_ showed opposite trend. However, we didn’t find a close linear relationship between A_max_ and leaf nitrogen content, both in the terms of N_a_ and N_m_ (Figure 3). Previous studies showed that under high irradiance, the photosynthetic rate is co-regulated more by investments of leaf N to carboxylation and electron transport (i.e., P_R_ and P_B_) compared with low irradiance (Hikosaka and Terashima 1996). In the present study, the three Rhododendron species studied had relative constant P_R_ and P_B_ values, but *R. delavayi* and *R. irroratum* had higher A_max_ and thus effectively enhance PNUE (Table 4). Efficient nitrogen use in photosynthetic machinery in accordance with the environment may enhance the fitness of these species in infertile habitats (Hassiotou et al. 2010). These results may imply that *R. delavayi* and *R. irroratum* have a broader ecological niche than *R. yunnanense*, however, leaf N content and leaf N reorganization did not meaningfully influence their physiological performance.

### Correlation between leaf structure and physiological function

The differences in photosynthetic capacity reflected the differences in leaf anatomy, physiology, and biochemistry. Stomata can effectively regulate gas exchange where water vapor exits the plant and CO_2_ enters. Leaf conductance, photosynthetic carbon gain and the potential transpiration rate are primarily determined by both stomatal aperture and density (Brodribb and Jordan 2011; Büssis et al. 2006). The stomata of *R. delavayi* and *R. irroratum* were more densely packed and smaller than *R. yunnanense*, and as a result, stomatal conductance and transpiration rate were nearly twice that of *R. yunnanense* (Table 3). As the ability of leaves to quickly open stomata and increase conductance may allow greater rates of carbon fixation to occur, so densely and small stomata partly contributed to larger stomatal conductance, and consequently higher photosynthetic rate of *R. delavayi* and *R. irroratum*.

Leaf structure as indicated by LMA was also positively correlated with G_s_ and photosynthetic capacity of the three *Rhododendron* species. The plant with higher LMA usually had greater surface area of leaf chloroplast (S_mes_) or mesophyll cells facing intercellular air spaces per area (S_c_), and consequently, mesophyll conductance and photosynthetic capacity (Terashima et al. 2001; Oguchi et al. 2003; Piel et al. 2002). Our study demonstrates that high *S*_mes_ and *S*_c_ were mainly due to thick palisade tissue. Notably, palisade thickness along with *S*_c_ may enhance the capture of photons on an area basis. Furthermore, the high *S*_c_ can enhance the diffusion of CO_2_ from the surface of mesophyll cells to chloroplasts, resulting in the positive correlation between *S*_c_ and *A*_max_ (Oguchi et al. 2005). However, other studies have suggested that high LMA has been associated with low mesophyll conductance, which can restrict the rate of CO_2_ assimilation (Terashima et al. 2006; Hassiotou et al. 2009). The negative effect of LMA on gas exchange can arise from changes in internal anatomy and an increase in the diffusion resistance of CO_2_ from the substomatal cavity to the chloroplasts (Niinemets 1999). Leaf surface properties including wax layers, epidermal cell shape, cuticular thickening, trichomes, mesophyll cell wall thickness and stomatal crypts contribute to high LMA, and can alter leaf structural properties and thus influence gas exchange (Terashima et al. 2005; Terashima et al. 2006).

Changes in LMA are caused by variations in internal anatomy, and there may also be secondary effect on foliar N content or N allocation to the photosynthetic machinery (Garnier and Laurent 1994). High LMA is often associated with more structural tissue and lower nitrogen content, but whether this is simply due to ‘dilution’ by the presence of more structural tissue, or also applies to the photosynthetic apparatus is not well understood (Hassiotou et al. 2010; Hikosaka 2004). In the present study, a negative linear relationship between LMA and N_m_ and a positive relationship between LMA and A_max_ were found in three *Rhododendron* species, but N content didn’t show significantly correlation with photosynthetic capacity either base on leaf area or leaf mass (Figure 3). The results suggest that more structural tissue in the three *Rhododendron* species, resulted lower mass-based nitrogen content, and changes in leaf photosynthesis, but importantly, these results did not arise from disparate N partitioning within leaves. However, in a comprehensive study of 25 species covering a 10-fold range in LMA, Harrisonlk et al. (2009) showed that the fraction of nitrogen allocated to cell walls is independent of LMA, and the relationship between the fraction of nitrogen allocated to Rubisco and LMA is curvilinear. These relationships between leaf N and leaf structure may arise because under field conditions, differences in stomatal conductance may dominate photosynthetic functioining, whereas differences in N content and internal leaf anatomy have only a marginal effect on photosynthetic functioning Mediavilla et al. (2001).

## Conclusion

In conclusion, three *Rhododendron* species exhibited significant differences in leaf anatomical and physiological characteristics related to their natural habitats. When compared with *R. yunnanense,* the divergence in leaf anatomical structures and physiological functioning of *R. delavayi* and *R. irroratum* reflected stronger ecophysiological performance to a higher light and drier environment. Variation in leaf photosynthesis across species was associated with variation in LMA, but not leaf nitrogen.

## Authors’ original submitted files for images

Below are the links to the authors’ original submitted files for images. Authors’ original file for figure 1 Authors’ original file for figure 2 Authors’ original file for figure 3 Authors’ original file for figure 4
