# Supplementary figures and images for: How do leaf anatomies and photosynthesis of three Rhododendron species relate to their natural environments?
Source: Bot Stud. 2014 Mar 20;55:36. doi: 10.1186/1999-3110-55-36 (PMC5432949; doi:10.1186/1999-3110-55-36)

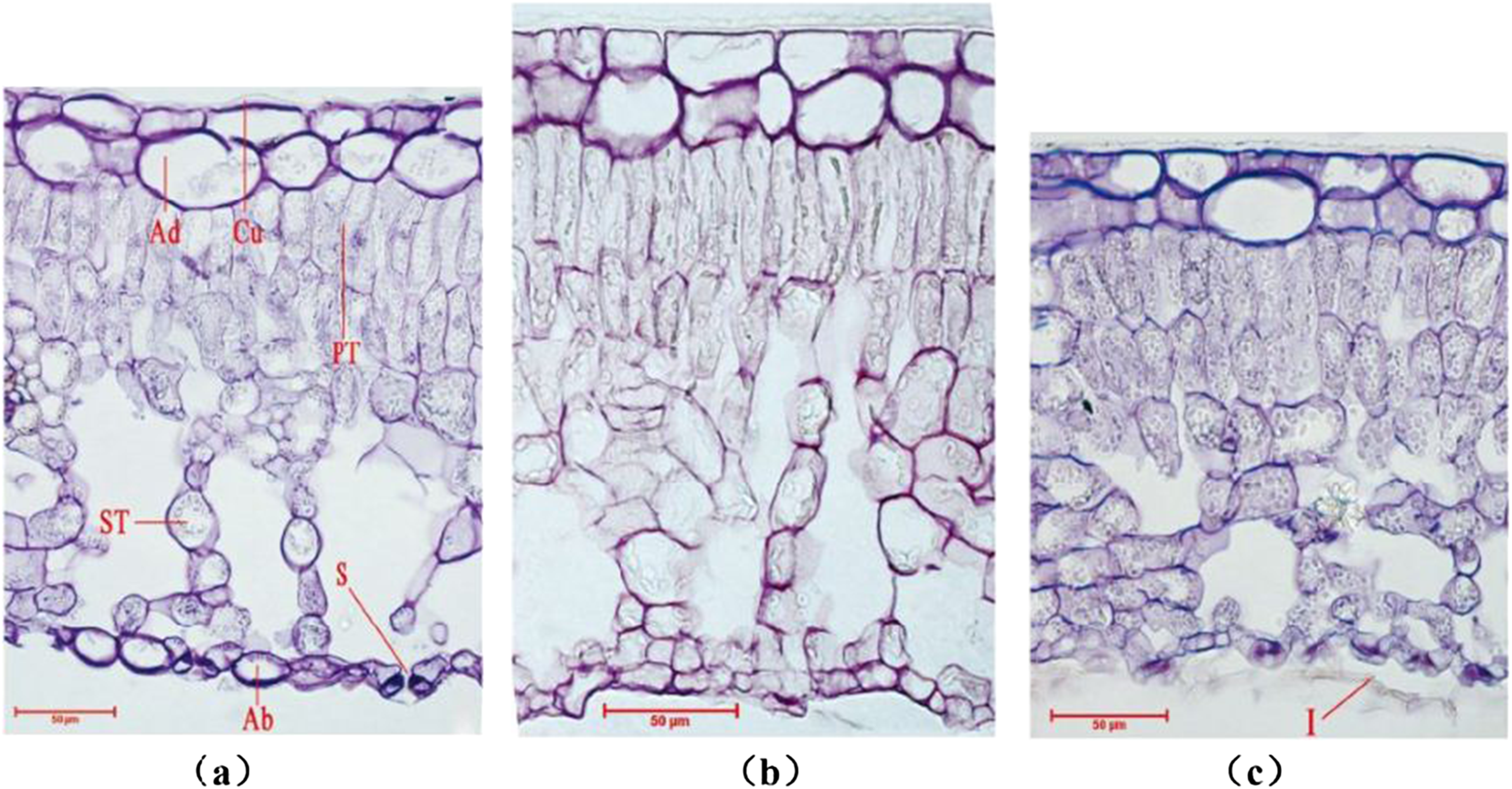

Supplement: Supplementary file 1 — Authors’ original file for figure 1 [file 40529_2012_82_MOESM1_ESM.tif]

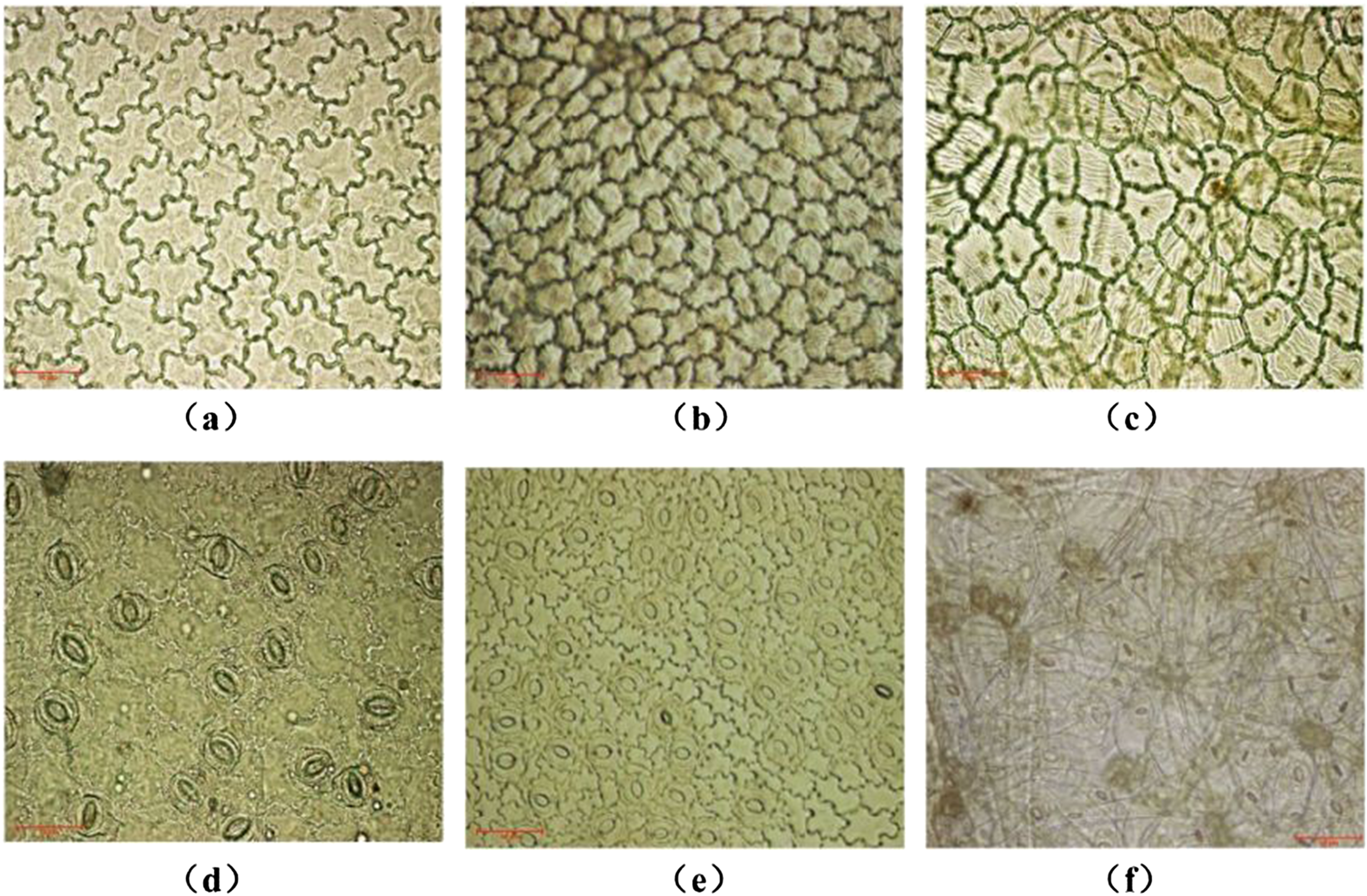

Supplement: Supplementary file 2 — Authors’ original file for figure 2 [file 40529_2012_82_MOESM2_ESM.tif]

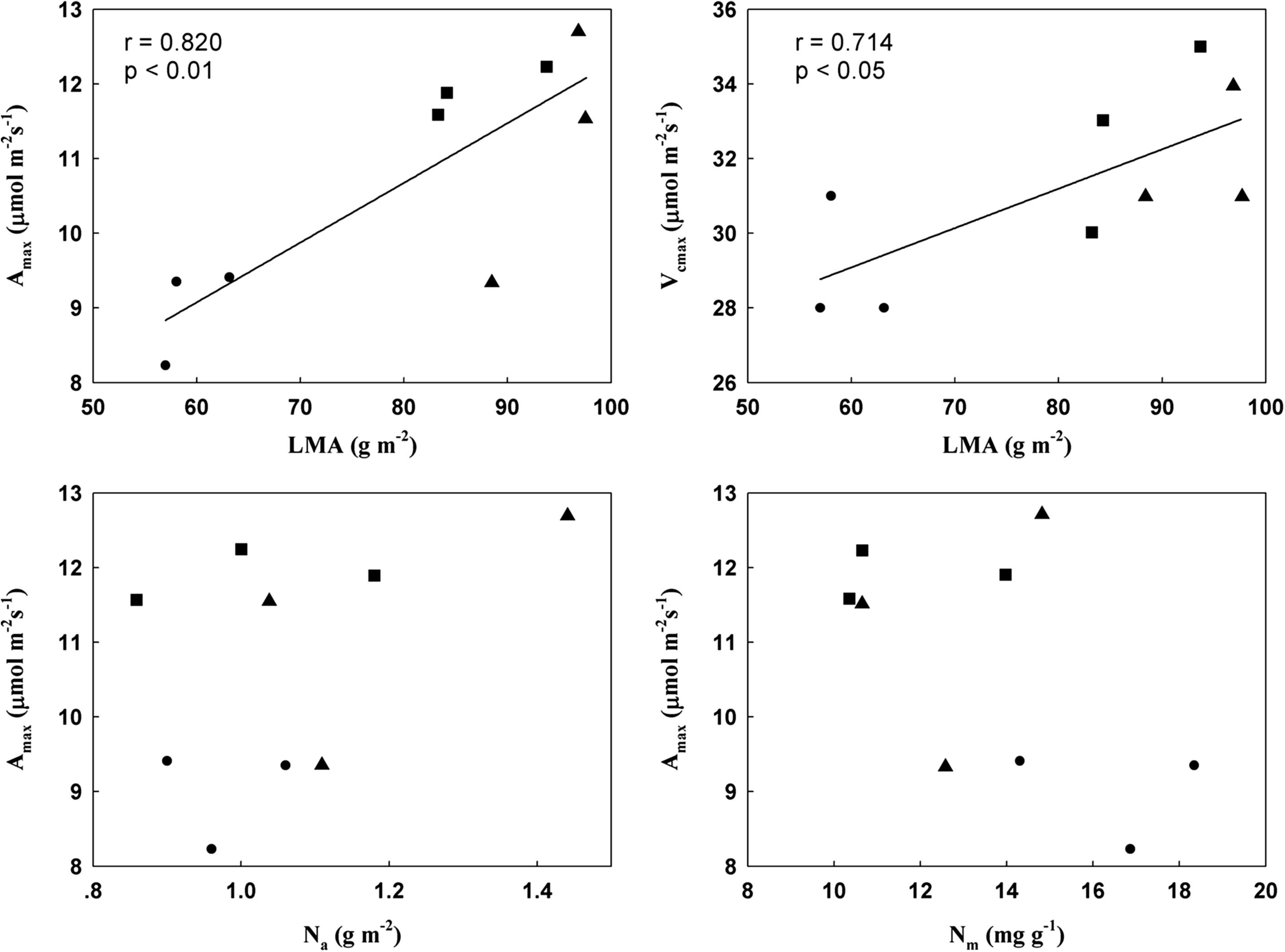

Supplement: Supplementary file 3 — Authors’ original file for figure 3 [file 40529_2012_82_MOESM3_ESM.tiff]

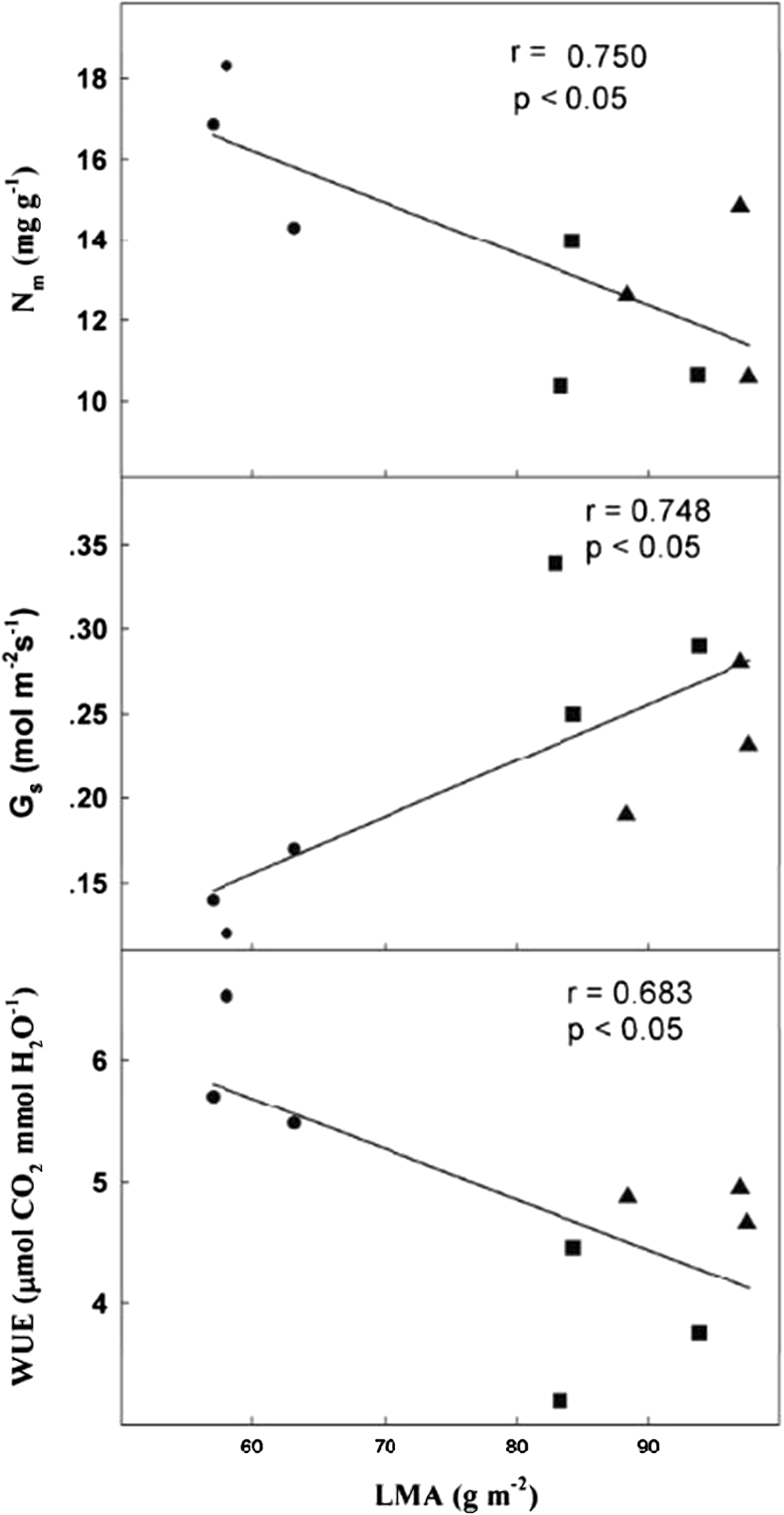

Supplement: Supplementary file 4 — Authors’ original file for figure 4 [file 40529_2012_82_MOESM4_ESM.tif]
